# Young Love: Romantic Concerns and Associated Mental Health Issues among Adolescent Help-Seekers

**DOI:** 10.3390/bs6020009

**Published:** 2016-05-06

**Authors:** Megan Price, Leanne Hides, Wendell Cockshaw, Aleksandra A. Staneva, Stoyan R. Stoyanov

**Affiliations:** 1 Institute of Health and Biomedical Innovation (IHBI), School of Psychology and Counselling, Faculty of Health, Queensland University of Technology, Brisbane 4059, Australia; leanne.hides@qut.edu.au (L.H.); w.cockshaw@qut.edu.au (W.C.); aleksandra.staneva@qut.edu.au (A.A.S.); stoyan.stoyanov@qut.edu.au (S.R.S.); 2 Young and Well Cooperative Research Centre, Melbourne 3067, Australia

**Keywords:** adolescen, youth, romance, mental health, suicide, self-harm, self-injury, help-seeking, relationship, breakup

## Abstract

Over 50% of young people have dated by age 15. While romantic relationship concerns are a major reason for adolescent help-seeking from counselling services, we have a limited understanding of what types of relationship issues are most strongly related to mental health issues and suicide risk. This paper used records of 4019 counselling sessions with adolescents (10–18 years) seeking help from a national youth counselling service for a romantic relationship concern to: (i) explore what types and stage (pre, during, post) of romantic concerns adolescents seek help for; (ii) how they are associated with mental health problems, self-harm and suicide risk; and (iii) whether these associations differ by age and gender. In line with developmental-contextual theory, results suggest that concerns about the initiation of relationships are common in early adolescence, while concerns about maintaining and repairing relationships increase with age. Relationship breakups were the most common concern for both male and female adolescents and for all age groups (early, mid, late adolescence). Data relating to a range of mental health issues were available for approximately half of the sample. Post-relationship concerns (including breakups) were also more likely than pre- or during-relationship concerns to be associated with concurrent mental health issues (36.8%), self-harm (22.6%) and suicide (9.9%). Results draw on a staged developmental theory of adolescent romantic relationships to provide a comprehensive assessment of relationship stressors, highlighting post-relationship as a particularly vulnerable time for all stages of adolescence. These findings contribute to the development of targeted intervention and support programs.

## 1. Introduction

Adolescence is associated with many psychosocial and developmental challenges, including the processing of intense emotions and “first loves” [1]. There is a growing body of work documenting the normative and salient nature of adolescent romance, as well as the behavioural, emotional and psychosocial sequelae of the experience [2,3,4,5,6]. It is now well evidenced that adolescent romance is an important developmental marker for adolescents’ self-identity, functioning and capacity for intimacy [4,7,8,9,10].

There have been a number of important theoretical contributions to the understanding of romantic relationships, from early through to late adolescence and the transition to young adulthood [7,9]. Approaches include evolutionary theories related to neuroendocrine functioning and genetics [11,12] and interpersonal theories which emphasize the nature and processes of changes in adolescents’ social relationships and their effect on cognitions, emotions and behaviours [7]. Theories of attachment [13], ego formation and psychosocial development [14] have been particularly influential.

Adolescent romance typically begins as brief relationships in early adolescence, progresses into sexual relationships in mid-adolescence (14–15 years) and onto more intense, committed relationships during later adolescence (16–18 years) [2,15,16]. Developmental-contextual theories of adolescent romantic stages also provide a framework for how romantic relationships assist young adults with addressing their identity and intimacy needs. Connolly and colleagues propose a framework containing four stages of romantic relationships [17]: (1) the *infatuation stage*—a pre-relationship stage, where young teens have the opportunity to explore their romantic passions through physical attraction on a personal level, without engaging a prospective partner; (2) *affiliate romantic stage*—a pre-relationship stage that typically occurs in a larger group context where an acknowledged couple relationship is not yet formed, rather, a decision is made whether or not to attempt entering into a relationship; (3) *intimate stage*—representative of a formed romantic couple; and (4) *committed* *stage*—where an established relationship borrows features resembling a marital relationship and a mutual commitment. According to this model, the evolution of adolescent romantic involvement is highly sensitive to the peer context and corresponds with the course of individual identity development [18].

Despite the brevity and reduced intimacy of relationships in early adolescence, reports of feelings of “love,” convictions of knowing the true nature of “love,” and feelings of confusion and hurt as a result of “love” have been documented in early adolescence and children as young as nine [1,18,19,20]. While these early attachment relationships may be unreciprocated and/or driven more by social than intimate factors, findings suggest the importance of acknowledging and examining romantic stressors across all developmental stages of adolescence [21].

Data from both Australian and international youth counselling services report romantic relationship concerns are one of the most common reasons young people seek counselling support [22]. Romantic relationships have been found to impact on psychosocial development and mental health during adolescence. For example, frequent or early dating and dating multiple partners has been linked with behavioural issues, poorer academic performance and employment prospects [10], and increased delinquency [23,24]. Similarly, several studies have found elevated levels of stress, anxiety and depressive symptoms among adolescents who engaged in romantic experiences compared to those who did not [25,26,27,28]. The breakup stage of a romance has also been specifically examined, revealing links to heightened likelihood of first onset major depressive disorder among older adolescents [29]. However, little research has examined the association between breakups and poor mental health in earlier stages of adolescence.

A number of variables related to adolescent romance have been associated with the risk of suicide attempts or completions in adolescents. These include incongruent partnership role-identities [30], negative sexual experiences [31] and stressful events including breakups [32] and relationship disputes [33]. Nevertheless, the extent to which romantic relationship issues contribute to suicide risk remains unknown. For example, one study found 76% of suicide attempts among adolescents who presented at an inner-city emergency department were related to relationship disputes, including peer, family and romantic relationships [33]. The present study sought to build on extant research by reporting rates of suicidal ideation (and self-harm) associated with romance specifically, including each of the discrete stages of romance, across early, middle and late adolescence.

Most research on adolescent romance has either looked at romantic involvement as a dichotomous variable (e.g., have or have not previously engaged in romance), focused on the various characteristics of dating (e.g., frequency and age of initiation) or focused on one distinct stage of the relationship, predominantly dissolution. Few studies have examined how age, gender and stages of romantic relationships may increase vulnerability to mental health issues. In one of few studies to date, Nieder and Seiffge-Krenke (2001) investigated the influence of relationship stages on stress levels in adolescents (*N* = 107) aged 14 to 17 years. They found the initiation and maintenance stages of relationships (e.g., not having a boyfriend or girlfriend, jealousy and developing and nurturing an equal and balanced relationship) were major causes of stress. Clear age differences in the level of stress associated with adolescent romance have also emerged. Two longitudinal studies report that the highest stress levels occur at the age of 14 and then decline with age [27]. However, small samples (*i.e*., *n* < 200) and gaps in the coverage of relationship stages [26,34] leave opportunity to improve understanding of associations between stressors of romance and mental health outcomes across relationship stages.

Gender may also be an important determinant of vulnerability to mental health issues related to relationship concerns, although its impact remains unclear [35,36]. Some research suggests that female adolescents are more likely to date [37] or become emotionally involved [38], and have an increased capability for developing and maintaining romantic relationships compared to their male peers. Girls with early-onset puberty are more likely to enter into sexual relationships with older boys, to experience more psychological distress during their early teens, and to engage in risk-taking behaviours such as drug and alcohol consumption than their peers [39]. Whether these characteristics result in the higher levels of interpersonal stress [40] and depression [20] among female daters compared to males is unknown. Other studies have found no gender differences in adolescents’ level of dating involvement and its impact on their psychosocial functioning [24].

### The Current Study

This paper examined records of 4019 counselling sessions with adolescents (10–18 years) seeking help from a national youth counselling service for a romantic relationship concern to explore: (i) what types and stage (pre, during, post) of romantic concerns adolescents seek help for; (ii) how they are associated with mental health problems, self-harm and suicide risk; and (iii) whether these associations differ by age and gender.

## 2. Materials and Methods

### 2.1. Participants

Participants were young people who sought support from a youth counselling service (Kids Helpline) between January 2013 and December 2013 and reported a romantic relationship concern. Kids Helpline is a free, Australian national counselling service (primarily funded by yourtown), which provides 24−7 counselling support to young people aged 5 to 25 years. During 2013, the Kids Helpline dataset captured the details of 72,416 counselling sessions with young people, including 45,176 sessions with adolescents aged 10–18 years [31]. Support was provided via telephone, email and real-time web counselling. Further details are provided in Table 1.

### 2.2. Data Source

The Kids Helpline is primarily funded by yourtown, an Australian Non-Government Organisation providing a variety of youth services. Kids Helpline is Australia’s only free, confidential telephone and online counselling service which provides 24−7 counselling support. Young people’s requests are handled by qualified counsellors, social workers, or psychologists who also undergo specific Kids Helpline training. Counsellors screen clients for suicidal thoughts and self-harm behavior, and assess their background and mental health during the counselling session in order to provide adequate and culturally appropriate counselling service. Assessment is based on self-report of existing diagnosis or counsellor expertise. Counselling sessions are regularly monitored by counselling supervisors and counselling advice or referral to another counsellor is provided when necessary. All data for this study were drawn from the Kids Helpline counsellor contact database, which captures non-identifying information on counselling sessions. It is completed at the end of each session by the counsellor as part of the services’ standard operational practice.

### 2.3. Sample Selection

The dataset comprises 38 fields for logging data, including ten mandatory fields. Mandatory fields include the date, time, length of session, client’s cultural background and frequency of contact, the nature of the client’s main concern (hereafter referred to as “primary concern”), any secondary concerns, referrals provided, whether the client presented with mental health issues, and thoughts of suicide or self-harm issues. The amount of information captured for non-mandatory items (including age and gender) varies depending on the privacy and confidentiality wishes of each client, the sensitivity of the concern (*i.e.*, the nature of some contacts is such that direct information gathering is either contraindicated or proves difficult) and the length of the counselling session.

Recording the nature of the client concerns is done by first categorising the problem from a set list of 12 categories. More specific detail about the concern is then recorded by selecting from a pre-defined list of problem-specific descriptors. All counsellors undergo training on the definitions of each problem category to ensure consistent understanding and reporting. Mandatory reporting requires selection of at least one problem classification per counselling session, with the option to report up to four problems per counselling session. Each counselling session is recorded as a separate contact resulting in some instances where multiple contacts correspond to one client.

### 2.4. Measures

#### 2.4.1. Demographic Variables

Age was recorded by counsellors then recoded into bands that broadly reflect key developmental stages of adolescence to allow for analysis between these stages, following established guidelines [4]. The three age bands were 10 to 14 years (early adolescence), 15–16 years (mid adolescence), and 17–18 years (late adolescence). Gender was recorded by counsellors as one of three options (“Male”/”Female”/”Unknown”). “Unknown” gender reports are the result of one of three reasons, as described under “Sample Selection.”

#### 2.4.2. Nature of Romantic Concern

The nature of the romance-related concern was assessed by counsellors and classified into one of eight categories (see Table 2). For the purpose of this study these categories were aggregated into three overarching relationship stages to reflect the three main relationship/developmental stages [17,18] (Table 2). Descriptive names were then given to the three relationship stages (*Initiation* for the pre-relationship stage; *Maintenance* for in-relationship stage; and *Dissolution* for the post-relationship stage). Initiation relates to seeking information about dating and initiating contact and corresponds to the affiliate romantic stage. The second stage, maintenance, corresponds to the intimate romantic stage in which a dyad is formed and assumes a more central role in structuring social interactions. Finally, dissolution, relates to the post-relationship stage where a commitment to a more stable relationship has not occurred.

#### 2.4.3. Mental Health

Mental health functioning was assessed by counsellors during the counselling session and classified as Yes/No/Unknown to indicate whether a client was likely to have a mental health issue. An affirmative assessment of mental health is defined by Kids Helpline as representing those clients who disclosed a previously diagnosed mental health disorder or illness or the counsellor assessed the presence of significant mental health symptomology consistent with one or more mental health disorders. This includes physical (e.g., poor sleep), emotional (e.g., overwhelming panic), behavioural (e.g., compulsions) and cognitive (e.g., disorganised thoughts) symptoms. Counsellors are not expected to formulate a diagnosis as part of their assessment nor is it a requirement to report specific details of any symptoms presented. An “unknown” assessment may represent one of several things: (1) the counsellor did not see a need to directly ask the client about this (most likely if no symptoms presented); (2) the counselling session was too short for the counsellor to confidently make an assessment; (3) gathering the information was contraindicated or proved difficult; or (4) there were client privacy or confidentiality concerns.

#### 2.4.4. Suicide Risk and Self-Harm

Risk of suicide and self-harm were also assessed by counsellors. Similar to mental health assessment, these variables were recorded using a three-point scale of Yes/No/Unknown. An affirmative assessment for suicide risk was recorded if the client disclosed that they were experiencing suicidal thoughts and further assessment by the counsellor verified the presence of a risk. An affirmative assessment of self-harm was applied if the client disclosed they had self-harmed in the few days or weeks prior to the counselling session and/or were struggling with the urge to injure themselves. Self-harm is defined by Kids Helpline as deliberate, non-life threatening, self-effected bodily harm with the intent of causing physical harm to themselves in ways that are not intended to end their own lives.

### 2.5. Ethics Approval

This study was ruled exempt from requiring ethical approval by the Queensland University of Technology Human Research Ethics Committee on the basis that the research was of negligible risk, involved the use of an existing dataset and only contained de-identified data collected as part of routine clinical practice at Kids Helpline.

### 2.6. Data Analysis

Associations between relationship stage (initiation, maintenance, dissolution) and age group (early, mid, late adolescence), mental health, self-harm and suicidal ideation were examined using two-tailed Pearson chi-square tests with the probability level set at *p* < 0.05. Effect sizes are expressed as Cramer’s V (or φ for 2 × 2 tests). To gain further insight, similar tests were performed for the eight specific relationship concern types. Associations of these variables with age as a continuous variable were analyzed with *t*-tests or one-way ANOVAs as appropriate.

## 3. Results

### 3.1. Sample Characteristics

A total of 4019 (8.7% of all concerns; primary reason for contact in 6.6% *N* = 3063) out of the 46,123 counselling sessions conducted with adolescents (10–18 years) seeking help from Kids Helpline between January and December 2013 reported a romantic relationship concern. The four other common reasons for seeking help were mental health and emotional wellbeing (24.9% *N* = 11,457), family relationships (13.3% *N* = 6134), suicide-related concerns (8.9% *N* = 4119) and peer relationships (7.9% *N* = 3644). In the present sample, 92 cases sought help due to concern for another person. These cases were excluded yielding a sample of 3927. Of these, 21.5% (*N* = 844) were male and 77.2% (*N* = 3032) were female, with gender not recorded for 1.3% (*N* = 51). Males reporting a romantic concern (age M = 16.41, SD = 1.49) were significantly older than females reporting a romantic concern (age M = 15.90, SD = 1.63), t(3874) = 8.21, *p* < 0.001. Methods of communication with clients were telephone (52.5%), real-time web-counselling (26.0%) and email (21.5%).

The presence or absence of a mental health issue as assessed by counsellors was recorded in 2014 cases. Of these, 36.8% (*N* = 742) were assessed to have a mental health issue. The presence or absence of a self-injury was recorded in 2000 cases. Of these, 22.6% (*N* = 452) were assessed as engaging in self-harm. The presence or absence of suicidal ideation was recorded in 2417 cases. Of these, 9.9% (*N* = 239) were assessed as engaging in suicidal ideation.

### 3.2. Romantic Relationship Concerns

#### 3.2.1. Relationship Stage by Age and Gender

**Figure 1:**
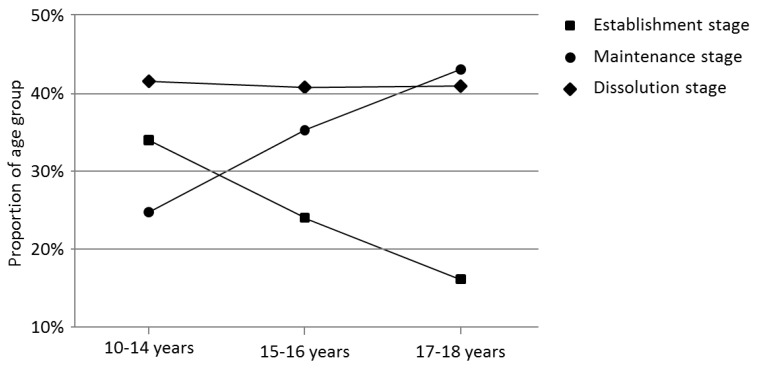
Developmental differences in stage for which telationship help is sought.

When the eight types of relationship concerns were aggregated into the three relationship stages, the most common stage was dissolution (40.9%) followed by maintenance (36.8%) and initiation (22.3%). Table 3 provides information on the age and gender characteristics for each area of romantic concern. A significant association between age and relationship stage was found (*F*_2, 3924_ = 66.40, *p* < 0.001). Mean ages were 15.50 (SD = 1.82), 16.28 (SD = 1.50) and 16.03 (SD = 1.55.) for establishment, maintenance and dissolution stages, respectively. Similarly, there was a significant association between age group (early, mid and late adolescence) and relationship stage χ2(4) = 120.69, *p* < 0.001, Cramer’s V = 0.12. Help-seeking for pre-relationship concerns reduced as adolescents got older, while help-seeking for ongoing relationship challenges and dissolution concerns increased with age (see Figure 1). Help-seeking for post-relationship issues was found to dramatically rise between early and mid-adolescence and then plateau, showing little difference between mid and late adolescence.

Gender was also significantly associated with relationship stage, although the effect size was small χ2(2) = 12.92, *p* = 0.002, Cramer’s V = 0.06. Males were significantly more likely than females to seek support regarding pre-relationship concerns (26.7% *versus* 21.0%) and less likely than females to seek support for post-relationship concerns (37.6% *versus* 41.9%). No gender differences in romantic concerns during a relationship were found.

#### 3.2.2. Specific Types of Concern by Age and Gender

Of the eight types of relationship concerns the most commonly reported were (7) relationship breakdown, (4) maintaining and sustaining relationships, and (3) wanting to establish a relationship, being recorded in 35.2%, 23.8% and 14.4% of cases, respectively. When types of concern were aggregated into the three relationship stages, the most common stage was dissolution (40.9%) followed by maintenance (36.8%) and initiation (22.3%). Table 3 provides further information on the age and gender characteristics for each specific type of romantic concern.

A significant association between the type of relationship concern and age was found, (*F*_7, 3919_ = 20.35, *p* < 0.001). Similarly, there was a significant association between romantic relationship stage and age group, χ2(4) = 120.69, *p* < 0.000 Cramer’s V = 0.12. Early adolescence was associated with an increased concern regarding establishment and a decreased concern regarding maintenance. Conversely, late adolescence was associated with a decreased concern regarding establishment and an increased concern regarding maintenance.

A significant association between gender and type of romantic concern was also found, χ2(7) = 42.11, *p* < 0.001, Cramer’s V = 0.10 (see Table 3). For most of the eight specific concern types, males showed greater concern in the establishment stage and females showed greater concern in the maintenance and dissolution stages. An exception was faithfulness concerns for which males showed the greatest concern. In all cases, however, effects were small.

### 3.3. Association between Romantic Concerns and Mental Health Problems

#### 3.3.1. Presence of Mental Health Issues

There was a significant association between relationship stage and mental health, χ2(2) = 50.64, *p* < 0.001, Cramer’s V = 0.16. Mental health issues were most prevalent in the dissolution stage (41.9%) followed by the maintenance stage (39.6%) and the initiation stage (22.5%). Rates of mental health issues did not differ between genders. There was, however, a significant association between mental health and age t(2012) = 8.70, *p* < 0.001. The mean age of those with an identified mental health issue (M = 16.46, SD = 1.43) was greater than that for those without (M = 15.84, SD = 1.43).

#### 3.3.2. Self-Harm

There was a significant association between relationship stage and self-harm, χ2(2) =44.69, *p* < 0.001, Cramer’s V = 0.15. Self-harm was most prevalent in the dissolution stage (28.4%) followed by the maintenance stage (22.2%) and the initiation stage (11.9%). There was also a significant association between gender and self-harm, χ2(1) = 34.68, *p* < 0.001, φ = 0.13, with females (25.9%) more likely than males (12.6%) to be experiencing self-harm issues. There was no significant association between age and self-injury. However, age was a significant factor when looking specifically at those seeking help regarding the dissolution stage t(839) = 3.60, *p* < 0.001. The mean age was lower for those engaging in self-harm (M = 15.70, SD = 1.59) than those who did not (M = 16.13, SD = 1.53).

#### 3.3.3. Suicide Risk

There was a significant association between relationship stage and suicidal ideation, χ2(2) = 43.95, *p* < 0.001, Cramer’s V = 0.14. Suicidal ideation was most prevalent in the dissolution stage (13.9%) followed by the maintenance stage (8.8%) and the initiation stage (3.5%). Rates of suicidal ideation did not differ between genders. Similarly suicidal ideation was not significantly related to age. This remained the case when looking specifically at those seeking help regarding the dissolution stage (see Table 4).

## 4. Discussion

This study aimed to explore the romantic concerns of adolescents seeking assistance from a youth counselling service, and determine associations between these concerns with age, gender and mental health outcomes. A unique dataset of 46,123 adolescent counselling sessions was analyzed. Romantic relationship concerns were the fifth most common reason for seeking help, representing 8.7% (*N* =4019) of all counselling contacts. This finding is consistent with previous research reporting romantic relationship concerns are one of the most common reasons for adolescent help-seeking [22].

Of the eight specific romantic concern types coded in the data set, breakups were identified as the most common, representing a third of all romance-related counselling sessions across both genders. Levels of concern with this specific concern were also consistent across age groups. The data therefore indicate that breakups are a common challenge for adolescents, with similar impact irrespective of age or gender.

With the exception of breakups, the developmental differences in help-seeking for romantic relationship issues identified in the current study are broadly consistent with developmental theories [3,5,16,22] and previous prospective research on the developmental stages of romantic stress in adolescence. Younger adolescents were more likely to seek help for concerns related to the initiation of relationships, when romance is typically a new experience and they lack skills and confidence in this area. Concerns about maintaining and sustaining relationships were more prominent in mid to late adolescence, when intimacy levels, emotional investment, skills and commitment in romantic experiences increase.

In counselling sessions where an assessment of the likely presence of a mental health issue was made, over a third (36.8%) involved a mental health issue. For cases where assessments were made, self-harm and suicidal ideation were recorded in 22.6% and 9.9% of sessions, respectively. While these rates are likely to be elevated due to the help-seeking nature of the sample, adolescents reporting dissolution stage issues (*i.e.*, breakup-related concerns; problems with the ex-partner), were significantly more likely to present with suicide and/or self-harm issues than those presenting with concerns about other relationship stages. Adolescents with dissolution stage issues were also significantly more likely to present with mental health issues compared to those concerned about establishment stage issues. These results are consistent with previous research finding that relationship breakups are associated with elevated levels of depression [18,20,21], anxiety [5] and stress [16].

These findings highlight the importance of exploring dissolution-stage issues in adolescents, including coping with and re-establishing life after a breakup and managing relationship difficulties with an ex-partner. Further research examining multiple risk and protective factors for mental health issues, suicide and self-harm among large samples of early, mid and late adolescents is required to better understand the impact of romantic relationships in both help-seeking and non-help-seeking youth.

The use of data on eight different types of romantic relationship concerns among a large geographical and ethnically diverse sample of help-seeking adolescents spanning early to late adolescence is a strength of this study. While 77.2% of help-seekers were female, no significant gender differences were found in the type of romantic concerns reported. A bias toward female help-seekers is also consistent with the characteristics of adolescent help-seekers to helplines [13]. The presence of mental health disorders, suicide and self-harm risk relied on self-report and the clinical assessments of counsellors with a range of levels of mental health training, rather than comprehensive mental health assessment with an established instrument. Help-seekers reporting romantic relationship issues as both a primary and secondary concern were included in the study, and it is unknown whether any of the concurrent mental health, suicide and/or self-harm issues reported were directly associated with the relationship issue or other additional concerns (e.g., family-related stress, peer-relationship issues) reported during their counselling session. Conclusions about the direction of the relationships reported in this study are also limited by the cross-sectional nature of the study. The limitations of the data collection system resulted in a substantial proportion of mental health, suicide and self-harm assessments marked as “unknown.” Improvements to Kids Helplines’ data reporting system to remove the use of “unknown” assessments were made subsequent to this study and are likely to benefit future research. Establishing mental disorder status using a standard instrument would also benefit future research. As data for this study draws on anonymous counselling contact records, it is not possible to differentiate between unique and repeat help-seekers; hence, reported statistics can only be equated to contacts rather than individuals. Finally, the use of a help-seeking cohort in this study limits the generalization of findings to non-help-seeking adolescents.

## 5. Conclusions

Despite its limitations, this study provides unique insights into the romantic concerns of adolescent help-seekers. Post-relationship concerns, especially breakups, present significant challenges for adolescents of all ages and genders, and had the strongest associations with concurrent mental health, suicide and self-harm risk. Together, these findings highlight the importance of supporting adolescents seeking help for romantic relationship concerns and the need to develop preventative and early intervention resources and programs aimed at increasing adolescents’ ability to cope with romance, particularly at the dissolution stage.

## Figures and Tables

**Table 1:** Romance help-seeking sample characteristics. *N* = 3927. Age *Mdn*: 16.0 (10–18).

	*N*	%
Gender: Female	3032	78.2
Aged 10–14	697	17.7
Aged 15–16	1493	38.0
Aged 17–18	1737	44.2
Location: Major city ^a^	1839	70.5
Australian Capital Territory	51	1.5
New South Wales	1212	35.3
Northern Territory	27	0.8
Queensland	664	19.4
Southern Australia	200	5.8
Tasmania	65	1.9
Victoria	983	28.7
Western Australia	229	6.7
Aboriginal or Torres Strait Islander/South Sea Islander	18	0.4
Living with parents or family	1264	87.2
Living with partner	67	4.6
Living out of home─not with a partner	80	5.6
Living with foster parents or services	38	2.7
Parents married	678	59.6
Parents separated	425	37.4
Both parents deceased	33	2.9
Employed full time	41	7.2
Employed part time	232	40.8
Not employed and not looking	205	36.1
Unemployed	75	13.2
Contacted service by email	836	21.3
Contacted service by telephone	2057	52.4
Contacted service by web counselling	1034	26.3
First contact to service	1272	32.4
Previous contact—No Electronic Case File	1016	25.9
Previous contact─Has Electronic Case File	1271	32.4
Previous contact─Has Electronic Case File plus Goal/Management/Crisis plan	142	3.6

All percentages are calculated on the basis of cases where information was available; ^a^ Based on postcode information provided by the client, then classified according to the Australian Bureau of Statistics’ ASGS system as either “major city”, “regional” or “remote”.

**Table 2:** Relationship-specific concern types and stages.

Relationship-Specific Categories Selected by Counsellors:	Relationship Stage
1. *Information seeking* (Seeking information about dating or romantic relationships)	Stage 1: Pre-relationship concerns *(Initiation stage)*
2. *Considering dating* (Considering whether to start dating)	
3. *Approaching* (Wanting to establish a relationship e.g., telling someone they like them)	
4. *Rupture and repair* (Maintaining and sustaining established relationships)	Stage 2: In-relationship concerns *(Maintenance stage)*
5. *Trust* (Specific faithfulness/“cheating“)	
6. *Considering ending* (Considering or wanting to end a relationship)	
7. *Breakup* (Relationship breakdown─coping with breakup or re-establishing life after)	Stage 3: Post relationship concerns *(Dissolution stage)*
8. *Dealing with ex* (Relationship difficulties with the ex-partner)	

**Table 3:** Romantic relationship concerns, by age and gender.

	Total		Age Group (years) N (%)						Gender *			
			10–14		15–16		17–18		Male		Female	
Total	3927	(100.0)	697	(17.7)	1493	(38.0)	1737	(44.2)	844	(21.8)	3032	(78.2)
Relationship stage 1: Establishment	875	(22.3)	236	(33.9)	359	(24.0)	280	(16.1)	225	(26.7)	636	(21.0)
Relationship stage 2: Maintenance	1445	(36.8)	172	(24.7)	526	(35.2)	747	(43.0)	302	(35.8)	1127	(37.2)
Relationship stage 3: Dissolution	1607	(40.9)	289	(41.5)	608	(40.7)	710	(40.9)	317	(37.6)	1269	(41.9)
Specific concern type 1:	177	(4.5)	39	(5.6)	68	(4.6)	70	(4.0)	39	(4.6)	136	(4.5)
Specific concern type 2:	131	(3.3)	35	(5.0)	53	(3.5)	43	(2.5)	22	(2.6)	107	(3.5)
Specific concern type 3:	567	(14.4)	162	(23.2)	238	(15.9)	167	(9.6)	164	(19.4)	393	(13.0)
Specific concern type 4:	934	(23.8)	108	(15.5)	353	(23.6)	473	(27.2)	193	(22.9)	729	(24.0)
Specific concern type 5:	262	(6.7)	32	(4.6)	90	(6.0)	140	(8.1)	69	(8.2)	191	(6.3)
Specific concern type 6:	249	(6.3)	32	(4.6)	83	(5.6)	134	(7.7)	40	(4.7)	207	(6.8)
Specific concern type 7:	1384	(35.2)	254	(36.4)	528	(35.4)	602	(34.7)	291	(34.5)	1076	(35.5)
Specific concern type 8:	223	(5.7)	35	(5.0)	80	(5.4)	108	(6.2)	26	(3.1)	193	(6.4)

Note: * Gender-specification was missing for 51 participants.

**Table 4:** Concurrent effects associated with relationship stages of concern, by age and gender.

	Relationship Concern	Total(%)		Affirmative Assessment		Age Group (years)						Gender ^a^			
						10–14		15–16		17–18		Male		Female	
Mental Health	Relationship stage 1: Establishment	440	(21.8)	99	(13.3)	12	(12.1)	33	(33.3)	54	(54.5)	19	(19.4)	79	(80.6)
	Relationship stage 2: Maintenance	739	(36.7)	293	(39.5)	12	(4.1)	101	(34.5)	180	(61.4)	76	(26.0)	216	(74.0)
	Relationship stage 3: Dissolution	835	(41.5)	350	(47.2)	47	(13.4)	125	(35.7)	178	(50.9)	79	(22.9)	266	(77.1)
	All Stages	2014	(51.4) ^b^	742	(100.0)	71	(9.6)	259	(34.9)	412	(55.5)	174	(23.7)	561	(76.3)
Self-harm	Relationship stage 1: Establishment	430	(21.5)	51	(11.3)	12	(13.2)	18	(10.8)	21	(10.8)	2	(3.6)	49	(12.4)
	Relationship stage 2: Maintenance	729	(36.5)	162	(35.8)	24	(26.4)	55	(32.9)	83	(42.8)	16	(28.6)	146	(37.1)
	Relationship stage 3: Dissolution	841	(42.1)	239	(52.9)	55	(60.4)	94	(56.3)	90	(46.4)	38	(67.9)	199	(50.5)
	All Stages	2000	(50.9) ^b^	452	(100.0)	91	(20.1)	167	(36.9)	194	(42.9)	56	(12.4)	394	(87.6)
Suicide	Relationship stage 1: Establishment	514	(21.3)	18	(7.5)	5	(14.7)	6	(7.2)	7	(5.7)	6	(9.0)	12	(7.1)
	Relationship stage 2: Maintenance	855	(35.4)	75	(31.4)	4	(11.8)	27	(32.5)	44	(36.1)	13	(19.4)	62	(36.5)
	Relationship stage 3: Dissolution	1048	(43.4)	146	(61.1)	25	(73.5)	50	(60.2)	71	(58.2)	48	(71.6)	96	(56.5)
	All Stages	2417	(61.5) ^b^	239	(100.0)	34	(14.2)	83	(34.7)	122	(51.0)	67	(28.3)	170	(71.7)

^a^ Numbers based on the percentage of available data. Missing data not calculated in total percentile; ^b^ Percentage of the total cohort (*N* = 3927) for whom an assessment was made.
